# Ventilatory Efficiency Is Reduced in People With Hypertension During Exercise

**DOI:** 10.1161/JAHA.121.024335

**Published:** 2023-06-22

**Authors:** Katrina Hope, Ben Chant, Thomas Hinton, Adrian H. Kendrick, Angus K. Nightingale, Julian F. R. Paton, Emma C. Hart

**Affiliations:** ^1^ Bristol Heart Institute CardioNomics Research Group, School of Physiology, Pharmacology and Neuroscience, Biomedical Sciences University of Bristol Bristol United Kingdom; ^2^ Department of Physiology, Faculty of Medical and Health Sciences The University of Auckland Auckland New Zealand; ^3^ Department of Respiratory Medicine University Hospitals Bristol National Health Service Foundation Trust Bristol United Kingdom; ^4^ Department of Cardiology Bristol Heart Institute, University Hospitals Bristol National Health Service Foundation Trust Bristol United Kingdom

**Keywords:** exercise, hypertension, minute ventilation/volume of expired CO_2_ slope, ventilatory efficiency, Hypertension, Physiology

## Abstract

**Background:**

An elevated ventilatory efficiency slope during exercise (minute ventilation/volume of expired CO_2_; V_E_/VCO_2_ slope) is a strong prognostic indicator in heart failure. It is elevated in people with heart failure with preserved ejection, many of whom have hypertension. However, whether the V_E_/VCO_2_ slope is also elevated in people with primary hypertension versus normotensive individuals is unknown. We hypothesize that there is a spectrum of ventilatory inefficiency in cardiovascular disease, reflecting an increasingly abnormal physiological response to exercise. The aim of this study was to evaluate the V_E_/VCO_2_ slope in patients with hypertension compared with age‐, peak oxygen consumption–, and sex‐matched healthy subjects.

**Methods and Results:**

Ramped cardiovascular pulmonary exercise tests to peak oxygen consumption were completed on a bike ergometer in 55 patients with primary hypertension and 24 normotensive controls. The V_E_/VCO_2_ slope was assessed from the onset of exercise to peak oxygen consumption. Data were compared using unpaired Student *t* test. Age (mean±SD, 66±6 versus 64±6 years; *P*=0.18), body mass index (25.4±3.5 versus 24±2.4 kg/m^2^; *P*=0.13), and peak oxygen consumption (23.2±6.6 versus 24±7.3 mL/min per kg; *P*=0.64) were similar between groups. The V_E_/VCO_2_ slope was elevated in the hypertensive group versus controls (31.8±4.5 versus 28.4±3.4; *P*=0.002). Only 27% of the hypertensive group were classified as having a normal V_E_/VCO_2_ slope (20–30) versus 71% in the control group.

**Conclusions:**

Ventilatory efficiency is impaired people with hypertension without a diagnosis of heart failure versus normotensive individuals. Future research needs to establish whether those patients with hypertension with elevated V_E_/VCO_2_ slopes are at risk of developing future heart failure.

**Nonstandard Abbreviations and Acronyms**
HFpEF: heart failure with preserved ejection fraction
VCO_2_
: volume of expired CO_2_
V_E_
: minute ventilation

**Clinical Perspective**

**What Is New?**

- Patients with primary hypertension (including patients with treated controlled blood pressure) without a diagnosis of heart failure have a higher minute ventilation/volume of expired CO_2_ slope than healthy controls.

**What Are the Clinical Implications?**

- This could signify early cardiac dysfunction and/or increased cardiac risk.
- Could this be used in the future for risk stratification and treatment intensification?

Hypertension affects 1 in 4 adults worldwide, and is a leading risk factor for future cardiovascular events and the development of heart failure (HF).^1^ Ventilatory efficiency, assessed via the relationship between ventilation and carbon dioxide production (minute ventilation/volume of expired CO_2_ [V_E_/VCO_2_]) during incremental exercise, is a noninvasive measure shown to reflect exercise indexes, such as cardiac output and pulmonary artery wedge pressure, that are normally obtained by invasive means.^2^

Poor ventilatory efficiency is a hallmark of HF with reduced ejection fraction and is predictive of future increased cardiovascular risk in patients diagnosed with HF with preserved ejection fraction (HFpEF)^3^ and a community population from the FHS (Framingham Heart Study) without HFpEF.^2^

The established normal range for V_E_/VCO_2_ in healthy subjects is 20 to 30.^4^ Current European Society of Cardiology Guidelines state that, in selected cases, V_E_/VCO_2_ may be used to aid in the diagnosis of HF with preserved ejection fraction (HFpEF)^5^; many of these patients have hypertension and need to be distinguished from those who have hypertension but no HF. However, no established cutoff is known as V_E_/VCO_2_ has not been investigated in patients with primary hypertension.

We hypothesize that there is a spectrum of ventilatory inefficiency in cardiovascular disease, which reflects an increasingly abnormal physiological response to exercise. We hypothesize that V_E_/VCO_2_ slope is higher in patients with hypertension compared with age‐, peak oxygen consumption–, and sex‐matched healthy subjects.

## Methods

The data that support the findings of this study are available from the corresponding author on request. Maximal cardiopulmonary exercise tests were completed at the University of Bristol (Bristol, UK) with a ramped 20‐ to 25‐W/min protocol on an upright cycle ergometer (Ergostik cardiopulmonary exercise testing system; Love Medical, UK). A total of 24 healthy, normotensive controls and 55 participants with primary hypertension (without HF; 40% treated controlled, 23.6% untreated, and 36.4% treated uncontrolled) were recruited and gave informed consent between 2016 and 2018. The studies were approved by UK National Health Service (NHS) Research Ethics Committees (16/SW/0004 and 15/SW/0030) and the Institutional Research and Innovation Department at the University Hospitals Bristol and Weston NHS Foundation Trust.

Blood pressure (BP) status was confirmed in all participants by 24‐hour ambulatory BP monitoring (Spacelabs), and hypertension was defined as either previous diagnosis of hypertension and prescribed antihypertensives or an average daytime ambulatory BP monitoring ≥135/85 mm Hg, as per 2011 National Institute for Health and Care Excellence guidelines.^6^

Peak exercise values are mean of the last 30 seconds of volitional exercise and V_E_/VCO_2_ slope calculated from linear regression of all breath‐by‐breath V_E_ and VCO_2_ values throughout exercise. BP was measured every 1 to 2 minutes throughout exercise with an automated cuff (Ergostik cardiopulmonary exercise testing system).

### Statistical Analysis

Unpaired Student *t* tests compared continuous variables. A 1‐way ANOVA with Dunnett multiple comparison tests compared V_E_/VCO_2_ slope among the normotensive and hypertensive subgroups (treated, untreated, or uncontrolled). Data are given as mean±SD. The α value was set at 0.05.

## Results

Hypertensive and normotensive participants were matched for age (66±6 versus 64±6 years; *P*=0.18), body mass index (25.4±3.5 versus 24±2.4 kg/m^2^; *P*=0.13), cardiovascular fitness (peak oxygen consumption; 23.2±6.6 versus 24±7.3 mL/min per kg; *P*=0.64), peak respiratory exchange ratio (1.23±0.1 versus 1.21±0.1; *P*=0.37) and male/female ratio (54%/46% versus 58%/42%). Daytime ambulatory systolic and diastolic BP levels were higher in hypertensive versus normotensive individuals (137±13 versus 121±10 mm Hg [*P*<0.0001] and 82±9 versus 74±6 mm Hg [*P*=0.0004], respectively). Peak exercise systolic and diastolic BP levels were higher in hypertension versus normotension (207±29 versus 178±27 mm Hg [*P*<0.0001] and 103±17 versus 88±11 mm Hg [*P*<0.0001], respectively), whereas peak heart rate was the same (143±18 versus 142±18 beats/min; *P*=0.9).

The V_E_/VCO_2_ slope was higher in the hypertensive compared with the normotensive group (31.8±4.5 [range, 20.16–43.17] versus 28.4±3.4 [range, 22.71–35.75]; *P*=0.002; Figure [A]). Peak breathing frequency was elevated in hypertensive individuals (34±8 versus 31±7 breaths/min; *P*=0.048; Figure [B]). However, peak end tidal carbon dioxide was lower in hypertensive versus normotensive individuals (36±3 versus 38±4 mm Hg; *P*=0.022; Figure [C]), with no difference in peak end tidal oxygen (116±4 versus 117±5 mm Hg; *P*=0.2892). When divided into subgroups, V_E_/VCO_2_ slope was higher in untreated (33.3±3.6; *P*=0.0027) and treated‐controlled (32.4±5.2; *P*=0.0050) hypertensive individuals versus normotensive individuals (28.4±3.4), whereas the treated‐uncontrolled group was not different (30.1±3.6; *P*=0.4429; Figure [D] and [E] for ambulatory systolic BP in all subgroups). The peak oxygen consumption was similar in all subgroups to normotensive individuals (Figure [F]).

**Figure:**
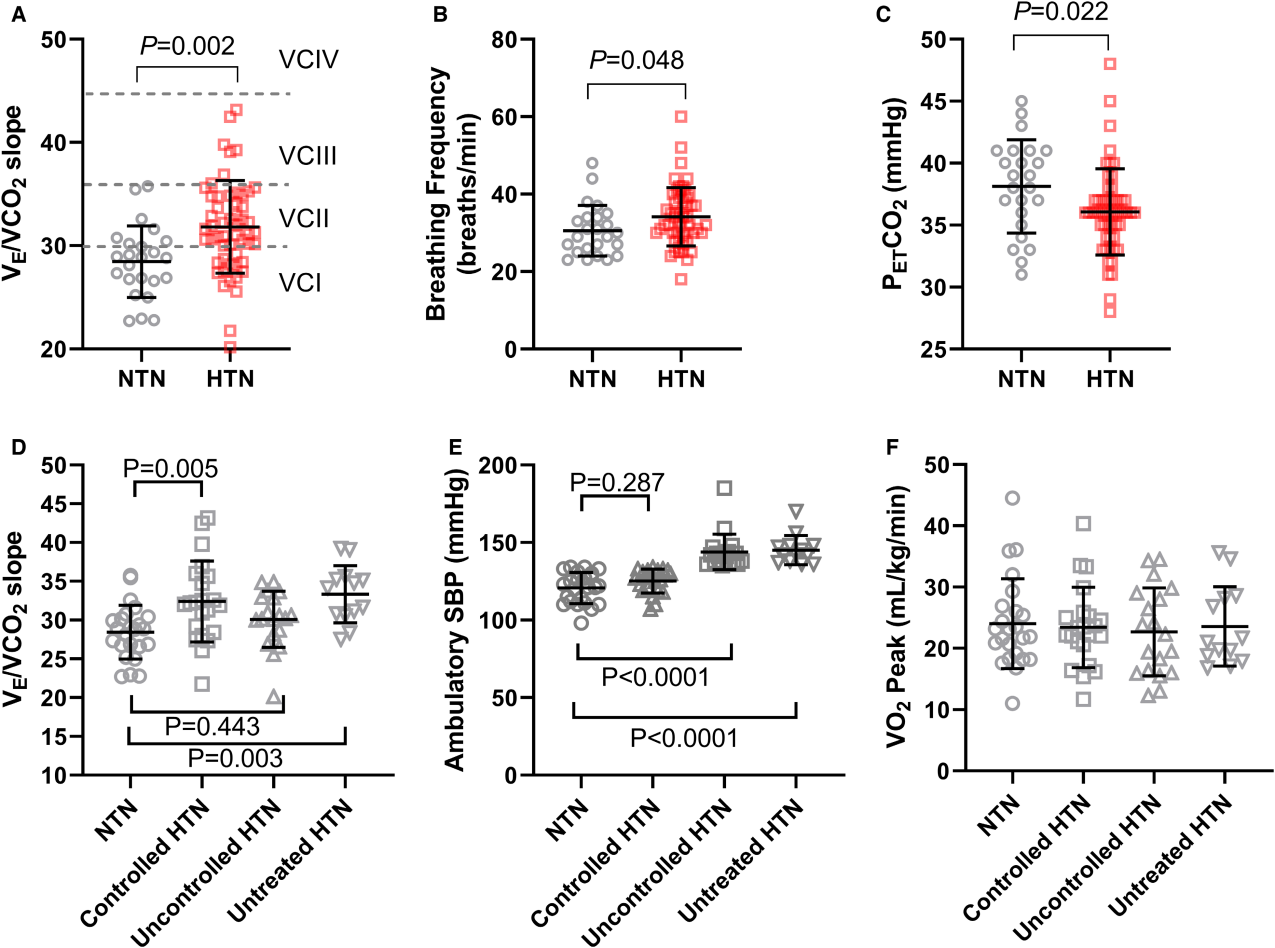
Comparison of exercise and ambulatory variables in normotensive (NTN) vs hypertensive (HTN) participants. **A** through **C**, HTN participants grouped together. **D** through **F**, HTN participants grouped by blood pressure control. Values are mean±SD, and *P* values are vs NTN participants. **A**, Minute ventilation/volume of expired CO_2_ (V_E_/VCO_2_) slopes in NTN vs HTN participants. The dotted lines divide the slopes into ventilatory classes (VCs) according to 2‐year cardiac event‐free survival in patients with heart failure, as discussed above^6^ (VCI, 97.2%; VCII, 85.2%; VCIII, 72.3%; and VCIV, 44.2%). **B**, Breathing frequency (breaths/min) at peak oxygen consumption (VO_2_). **C**, End‐tidal carbon dioxide (P_ET_CO_2_) at peak VO_2_. **D**, Comparison of V_E_/VCO_2_ slopes in subgroups of participants with HTN. Treated‐controlled HTN (n=22); treated‐uncontrolled HTN (n=19); untreated HTN (n=13); NTN (n=24). **E**, Ambulatory daytime systolic blood pressures (SBPs) in HTN subgroups vs NTN. **F**, Peak VO_2_ in HTN subgroups vs NTN.

## Discussion

Patients with hypertension have an elevated V_E_/VCO_2_ slope compared with age‐matched normotensive controls, indicating ventilatory inefficiency during exercise.

When grouping our participants according to a V_E_/VCO_2_ slope classification, suggested by Arena et al for HF,^7^ only 27.3% of participants with hypertension would be classified as having normal values (V_E_/VCO_2_ slope of 20–30), with 70.9% being in classes 2 (58.2% [range, 30–35.9]) and 3 (12.7% [range, 36–44.9]), indicating potential increased cardiovascular risk. This is compared with 70.8% of our normotensive individuals classified as having normal ventilatory efficiency. This suggests a degree of abnormal cardiopulmonary exercise response even in people with treated‐controlled hypertension, despite having a similar peak oxygen consumption to the control group. Because elevated V_E_/VCO_2_ slopes reflect the severity of disease in people with HF with reduced or even preserved ejection fraction,^7^, ^8^ potentially the elevated V_E_/VCO_2_ slope reflects some degree of cardiac dysfunction in patients with hypertension, which needs to be established. This is important because hypertension is a leading comorbidity associated with the development of HFpEF.^9^ Future research needs to determine whether an increased V_E_/VCO_2_ slope could be a signpost for identifying patients with hypertension at risk of development of future HFpEF and/or cardiac events, who may benefit from further investigation and treatment intensification. Further work is needed to understand the underlying cause of the elevated slope; is it ventilation/perfusion mismatching attributable to elevated pulmonary pressures, cardiac dysfunction, or ventilatory limitation?^10^ In addition, could the increased activity of the carotid chemoreceptors and peripheral metaboreceptors be contributing to ventilatory inefficiency?^11^, ^12^

## Conclusions

In our cohort of participants, we show that patients with hypertension (apart from the uncontrolled hypertensive group) have lower ventilatory efficiency in comparison to normotensive controls, as measured by V_E_/VCO_2_ slope. This reproducible, noninvasive measure may guide risk stratification and decision‐making with regard to antihypertensive treatment and prevention of cardiac events and HFpEF. Future research is needed to establish the exact mechanisms underlying the reduced ventilatory efficiency in hypertension, whether this is predictive of subsequent of future cardiovascular‐related hospitalization or HFpEF.

## Sources of Funding

This work was supported by the British Heart Foundation (FS/18/18/33522 [Dr Hinton], FS/17/49/32917 [K. Hope], and AA/18/7/34219 [Dr Hart]), National Institute for Health and Care Research Biomedical Research Centre, and the James Tudor Foundation. Dr Paton is funded by Health Research Council of New Zealand and the Sidney Taylor Trust. The views expressed in this article are those of the authors and not necessarily those of the institution or funders.

## Disclosures

None.
